# *Capnocytophaga ochracea* sialidase drives its biofilm maturation and host cell interactions

**DOI:** 10.3389/fcimb.2026.1841812

**Published:** 2026-06-19

**Authors:** Jing He, Shouliang Zhao, Yanqin Ju

**Affiliations:** 1 Department of Endodontics, Shanghai Ninth People’s Hospital, Shanghai Jiao Tong University School of Medicine, Shanghai, China; 2 College of Stomatology, Shanghai Jiao Tong University, Shanghai, China; 3 National Clinical Research Center for Oral Diseases, National Center for Stomatology, Shanghai, China; 4 Shanghai Key Laboratory of Stomatology, Shanghai, China; 5 Department of Stomatology, Huashan Hospital, Fudan University, Shanghai, China

**Keywords:** biofilm formation, *Capnocytophaga ochracea*, sialidase, early colonizer, host interaction

## Abstract

**Introduction:**

*Capnocytophaga ochracea*, an early colonizer of oral biofilms associated with gingivitis, plays an incompletely understood role in periodontal pathogenesis. While sialidases are established virulence factors in late-colonizing ‘red-complex’ pathogens, their function in early colonizers, such as *C. ochracea*, remains unknown.

**Methods:**

To address this knowledge gap, we identified and characterized Co-NanH, the sole sialidase of *C. ochracea*. We performed phylogenetic and structural analyses to compare it with known sialidases. Enzymatic assays were conducted using recombinant Co-NanH to determine its pH optimum, substrate specificity, and kinetic parameters. Furthermore, we utilized sialidase inhibitors (such as DANA) and generated a *nanH* genetic deletion mutant (*ΔnanH*) to assess the enzyme’s role in planktonic growth, biofilm formation, and interactions with human gingival epithelial cells (adhesion and internalization).

**Results:**

Phylogenetic and structural analyses revealed that Co-NanH shares significant homology with a sialidase from *Tannerella forsythia* but possesses a distinct Sec/SPI signal peptide and a monomeric structure. Recombinant Co-NanH exhibited optimal activity at pH 5.5 and cleaved both α2,3- and α2,6-linked sialic acids, with kinetic parameters comparable to those of red-complex pathogen sialidases. Inhibitors like DANA potently suppressed its activity and reduced *C. ochracea* biofilm formation. Crucially, genetic deletion of *nanH* abolished sialidase activity without affecting planktonic growth. The *ΔnanH* mutant exhibited severely impaired biofilm formation, characterized by reduced biomass, thickness, and initial attachment. Furthermore, the mutant showed significantly reduced adhesion to and internalization by human gingival epithelial cells compared with wild-type and complemented strains.

**Discussion:**

These findings establish Co-NanH as a key mediator of *C. ochracea* in biofilm maturation and host cell interactions, revealing a functional parallel between early colonizers and classical periodontal pathogens. This work provides functional evidence that sialidase activity in an early colonizer contributes to oral dysbiosis by facilitating biofilm development and priming the host environment for disease progression.

## 1 Introduction

The human oral cavity harbors a complex and dynamic microbial community, the architecture of which is critical for maintaining oral health. Dysbiosis within this polymicrobial biofilm can lead to the development of prevalent inflammatory conditions such as gingivitis and periodontitis (Manoil et al., 2024)*. Capnocytophaga ochracea*, a Gram-negative, anaerobic, rod-shaped bacterium, is a prominent member of the oral microbiota and is frequently isolated from subgingival plaque, particularly in individuals with gingivitis (Park et al., 2015; Idate et al., 2020; Iniesta et al., 2023; Li et al., 2023; Malan-Müller et al., 2024; Zhao et al., 2025). As a member of the ‘green complex’, *C. ochracea* is also recognized as an important early colonizer in the successional development of dental biofilms (Socransky et al., 1998). This role is further underscored by spatial analyses of oral biofilms, which reveal that *C. ochracea* populates a distinct, broad band at the biofilm periphery, a strategic location for interacting with both the host and later-colonizing pathogenic species (Mark Welch et al., 2016). This spatial positioning and association with disease suggest that *C. ochracea* plays a significant, yet incompletely understood, role in shaping the biofilm environment and potentially contributing to the pathogenesis of periodontal diseases.

The pathogenic potential of oral bacteria is often mediated by specific enzymatic activities that facilitate nutrient acquisition, biofilm development, and evasion of host immunity (Gendron et al., 2000; Baker et al., 2024). Among these, bacterial sialidases (neuraminidases), typically classified within glycoside hydrolase (GH) family 33 (Lipničanová et al., 2020), are of particular importance. These enzymes catalyze the cleavage of terminal sialic acid residues from host glycoconjugates. Elevated sialidase activity is a clinical hallmark of periodontal disease, detected in gingival crevicular fluid (GCF) from inflamed sites, implicating these enzymes in disease progression (Gul et al., 2016). Indeed, sialidases are well-established virulence factors for keystone pathogens of the ‘red complex’, including *Porphyromonas gingivalis*, *Tannerella forsythia* and *Treponema denticola*. In these organisms, sialidases contribute to diverse pathogenic processes, such as modulating biofilm architecture, host-pathogen interactions, and host immune responses. For instance, the *P. gingivalis* sialidase (PG0352) is involved in biofilm formation, capsule synthesis, serum resistance and its virulence (Li et al., 2012). The *T. forsythia* sialidase (Tf-NanH) enhances biofilm growth and interaction with human cells (Honma et al., 2011; Frey et al., 2018). Similarly, *T. denticola* sialidase (TDE0471) affects nutrient acquisition, complement activation, membrane attack complex deposition and pathogenicity (Kurniyati et al., 2013).

While sialidases were extensively studied in late-colonizing pathogens, their role in early colonizers such as *C. ochracea* is less clear. Sialidase activity has been documented in select human *Capnocytophaga* species, including *C. ochracea* and *C. sputigena (*Moncla et al., 1990). Furthermore, a sialidase (SiaC) from the canine-associated species, *C. canimorsus*, a zoonotic pathogen, has been characterized in detail. SiaC was shown to be a critical virulence factor, enabling the bacterium to deglycosylate host cell surfaces to acquire essential nutrients for growth (Mally et al., 2008; Mally et al., 2009; Renzi et al., 2011). However, despite these observations, the gene(s) encoding this activity in human-associated *Capnocytophaga* have not been identified, and no sialidase from these species has been biochemically or functionally characterized. The function of this enzymatic activity in an early colonizer—whether for nutrition, biofilm structuring, or priming the environment for subsequent pathogens—remains a critical open question.

In this study, we sought to address this knowledge gap by identifying and characterizing the sialidase from *C. ochracea.* We identified a putative sialidase gene in *C. ochracea* CCUG 9716^T^, which we subsequently cloned, expressed, and purified for detailed biochemical analysis. To elucidate its physiological function, we constructed a precise gene knockout mutant and a corresponding complemented strain. Using the genetic approach, we investigated the role of sialidase in biofilm formation and in mediating interactions with human gingival epithelial cells. We demonstrate that this enzyme is solely responsible for the sialidase activity in *C. ochracea* and is critical for biofilm maturation and the modification of host cell surface glycans, thereby providing the first functional characterization of a sialidase from a human oral *Capnocytophaga* species.

## 2 Materials and methods

### 2.1 Bacterial strains and growth conditions

*C. ochracea* CCUG 9716 wild type (WT), *nanH* gene deletion mutant (Δ*nanH*) and complemented (comΔ*nanH*) strains were cultured anaerobically (85% N_2_, 10% H_2_, 5% CO_2_) at 37 °C in the tryptic soy broth (TSB) supplemented with 5 μg/mL yeast extract, 5 μg/mL hemin, and 1 μg/mL vitamin K. The media for Δ*nanH* and comΔ*nanH* strains were supplemented with 10 μg/mL erythromycin or tetracycline, respectively. *Escherichia coli* strains DH10B and BL21 (DE3) were cultivated in Luria-Bertani (LB) broth or agar (Thermo Fisher Scientific), with the appropriate antibiotic selection.

### 2.2 Bioinformatic analyses

A representative sialidase sequence (SiaC) were utilized as the initial query for BLASTp searches against the *Capnocytophaga* genus. To refine detection, the catalytic domain of *C. ochracea* sialidase served as a secondary query. Identified sialidase sequences were downloaded from the NCBI database. Multiple sequence alignments (MSA) were constructed using MEGA11 (Tamura et al., 2021) and visualized via ESPript 3.0 (Robert and Gouet, 2014). Phylogenetic relationships among these sialidase amino acid sequences were inferred using the default Maximum Likelihood approach (Stamatakis et al., 2008) in MEGA11. The NCBI Conserved Domain Database was explored to confirm the presence of sialidase homologues and delineate the catalytic versus non-catalytic domains. Signal peptides and their cleavage sites were predicted using the SignalP 6.0 Webserver (Teufel et al., 2022).

### 2.3 Cloning, expression and purification of recombinant proteins

The nucleotide sequence encoding Coch_0016, excluding the secretion signal sequence, was PCR amplified from *C. ochracea* genomic DNA and cloned into pET28a (Novagen, Merck Millipore) via BamHI/HindIII. The plasmid details and primer sequence are listed in **Supplementary Tables 1** and **2**. The resulting plasmid, pET28a-Co-NanH, was verified by Sanger sequencing and routinely maintained in *E. coli* DH10B, cultivated in LB broth or LB-agar plates containing kanamycin (50 μg/mL).

For protein expression, pET28a-Co-NanH containing PCR inserts with the correct DNA sequences was transformed into *E. coli* BL21 (DE3). Cultures were grown in LB broth with 50 μg/mL kanamycin at 37 °C until the optical density at 600 nm (OD_600_) reached *ca.* 0.4-0.6. Protein expression was induced by adding 0.2 mM isopropyl β-D-1-thiogalactopyranoside (IPTG; GE Healthcare), and the cultures were further incubated at 25 °C with shaking at 200 rpm for 12 h. Cells were pelleted by centrifugation (6000 × g, 4 °C, 10 min), washed once with 30 mL pre-chilled phosphate-buffered saline (PBS; pH 7.4), and stored at -70 °C.

The recombinant N-terminally His_6_-tagged Co-NanH was loaded in a 5 mL HiTrap Chelating HP column (GE Healthcare) impregnated with nickel ions and purified by a linear gradient elution buffer (25 mM Tris-HCl, pH 7.4, 500 mM NaCl, 250 mM imidazole) on fast protein liquid chromatography using an ÄKTA purifier system (GE Healthcare). Purity was assessed via SDS−PAGE on 12% acrylamide/bis-acrylamide gels (37.5:1; Bio-Rad), and protein concentrations were determined using the BCA Protein Assay Kit (Thermo Fisher Scientific).

### 2.4 Enzymatic characterization of purified Co-NanH

#### 2.4.1 Optimal pH and reaction kinetics against MUNANA

Co-NanH activity was determined using 4-trifluoromethy-lumbelliferyl-d-N-acetylneuraminic acid (MUNANA, Sigma-Aldrich) in 96-well plates. 200 µL assay mixtures contained 200 nM enzyme, 0.1 mM MUNANA, 50 mM NaCl and the mixed buffer (50 mM Tris-HCl, Bis-Tris and sodium acetate). A microplate reader (SpectraMax^®^ M2e Multimode Microplate Reader) was used to continuously quantify sialidase activity by measuring 4-MU fluorescence (λ_ex/em_ = 360/460 nm). Using a standard curve of 4-MU fluorescence signal at defined concentrations, the rate of sialidase activity was calculated in Prism 10 and expressed as 4-MU release μmol. min^-1^. μmol^-1^. The mixed buffer (pH values: 4.3, 4.7, 5.1, 5.5, 5.9, and 6.3) was used to determine the optimal pH for Co-NanH. Kinetic parameters (*V*_max_, *k*_cat_ and *K*_M_) were obtained by fitting data to the Michaelis-Menten model, incorporating results from sets of assays performed in triplicate. 200 nM Co-NanH protein was incubated in the presence of a variable concentration of MUNANA (0-200 μM) in the mixed buffer at pH 5.5.

#### 2.4.2 Substrate specificity of Co-NanH with 3’-sialyllactose and 6’-sialyllactose

Substrate specificity was assessed using 3’-sialyllactose and 6’-sialyllactose (3’-SL, 6’-SL; Sigma-Aldrich). Sialidase activity was quantified by measuring the released sialic acid (Neu5Ac) using a modified thiobarbituric acid (TBA) assay. 50 µL reactions containing 50–100 nM Co-NanH and increasing concentrations of 3’-SL or 6’-SL in 50 mM sodium phosphate (Sigma-Aldrich), 200 mM NaCl, pH 7.4 were incubated at 37 °C and halted at 1, 3 and 5 min after initiation by adding 25 µL 20 mM sodium periodate (Sigma-Aldrich) in 60 mM H_2_SO_4_ (Sigma-Aldrich). The reactions were left to oxidize for 30 min at 37 °C. Oxidation was then halted by adding 20 µL 2% (w/v) sodium meta arsenite (Sigma-Aldrich) in 500 mM HCl. 47 μL of this reaction was added to 100 μL of 100 mM TBA (Sigma-Aldrich), pH 9.0, and incubated at 95 °C for 7.5 min, then centrifuged at 1,500 × g for 5 min, and the absorbance was quantified at 549 nm. A standard curve of Neu5Ac (Sigma-Aldrich) concentrations was used to calculate sialic acid release in Neu5Ac release μmol. min^-1^. μmol^-1^. After plotting Neu5Ac release under different concentrations of substrates (3’-SL or 6’-SL), *V*_max_, *k*_cat_ and *K*_M_ were determined as above for MUNANA.

#### 2.4.3 Inhibition effect of DANA, siastatin B and oseltamivir on Co-NanH activity against MUNANA

The inhibitory effect of N-acetyl-2,3-dehydro-2-deoxyneuraminic acid (DANA, Sigma-Aldrich), siastatin B (Sigma-Aldrich) or oseltamivir (Sigma-Aldrich) on Co-NanH was determined with the assay using MUNANA as the substrate. Briefly, 200 nM Co-NanH was incubated with 0.1 mM MUNANA in the same reaction buffer as described above in the presence of different concentrations (0 - 200 µM) of DANA, siastatin B or oseltamivir. Sialidase inhibition was expressed as the percentage of the reaction rate at a given inhibitor concentration, relative to the reaction rate in the absence of an inhibitor. Assays were performed in triplicate, with error bars shown.

### 2.5 Sialidase activities in *C. ochracea*, *P. gingivalis* and *T. forsythia*

*C. ochracea*, *P. gingivalis* and *T. forsythia* were anaerobically grown on blood agar for 3 days at 37 °C. Colonies were resuspended in fresh TSB and adjusted to approximately 2 × 10^6^ CFU/mL, respectively. Sialidase activity was quantified using MUNANA. Unless otherwise indicated, assays were performed in 200 μL reaction volumes containing 50 μL of bacterial suspension and 0.1 mM MUNANA in reaction buffer (pH 5.5, 50 mM NaCl). Fluorescence was monitored continuously in triplicate at 37 °C for 1–3 h using a microplate reader.

**Figure 1:**
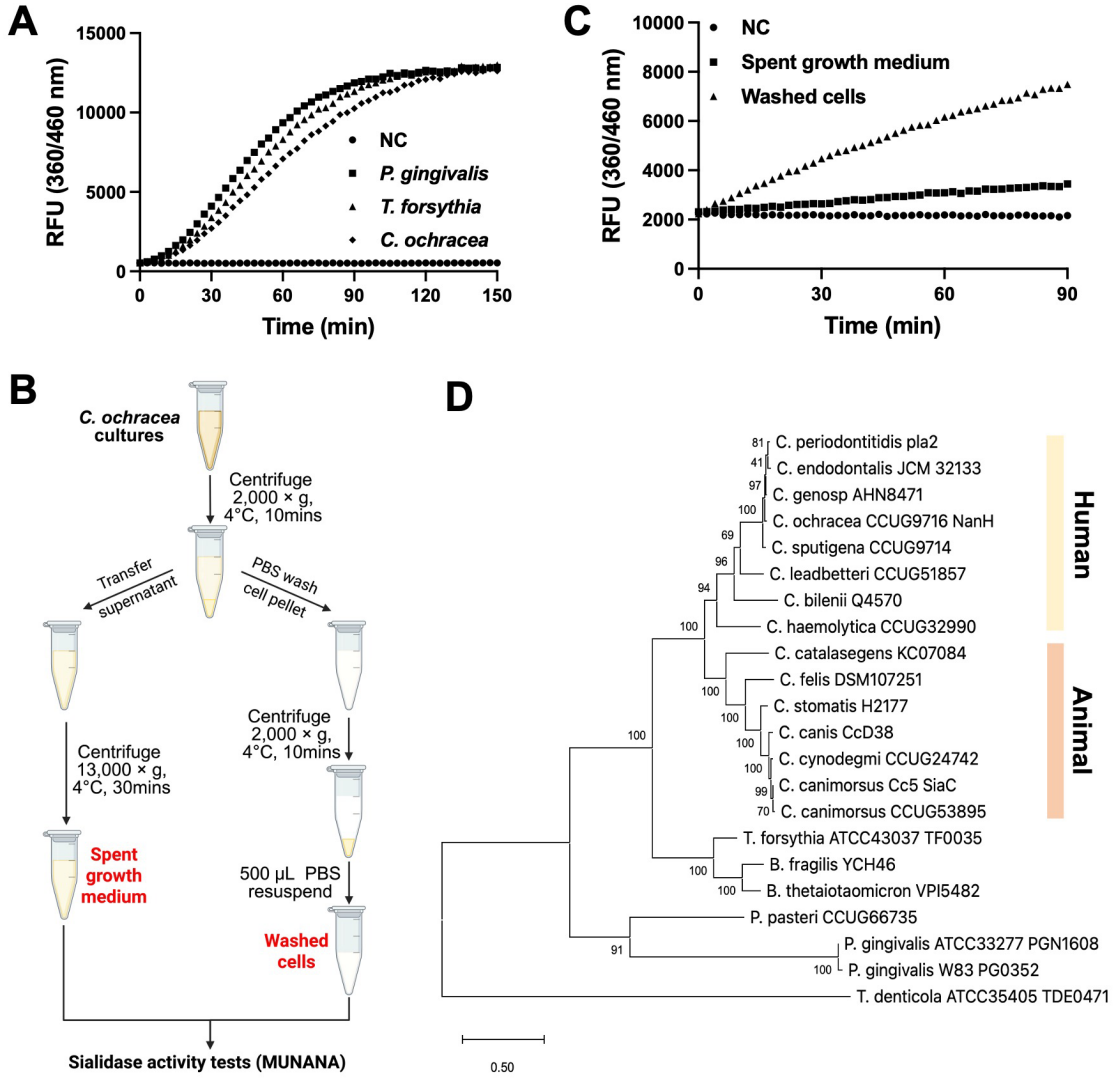
Sialidase activity in *C. ochracea* and phylogenetic analysis of the GH33 *exo-α*-sialidases encoded by selected species. **(A)** Sialidase activities in the cultures of *P. gingivalis*, *T. forsythia* and *C. ochracea* were detected using MUNANA. NC, negative control (fresh TSB). **(B)** Schematic diagram showing the workflow for obtaining the spent growth medium and washed cells from *C. ochracea*. **(C)** Sialidase activity in the spent growth medium and in washed cells of *C. ochracea* was detected using MUNANA. NC, negative control (fresh TSB). All values represent relative sialidase activity based on fluorescence units and do not reflect absolute sialidase protein concentrations in each fraction. **(D)** The Maximum Likelihood phylogenetic tree of sialidase homologues in *Capnocytophaga* and related species was constructed by MEGA 11 using the default settings. The bacterial strain and several sialidase designations are indicated.

To assess extracellular and cell-associated sialidase activity of *C. ochracea* (**Figure 1B**), 1 mL bacteria culture was centrifuged (4 °C, 2,000 × g, 10 min) to crudely separate the spent growth medium and cell pellet. The supernatant was clarified (4 °C, 13,000 × g, 30 min) to obtain the spent growth medium, while the pellet was washed once with 500 μL pre-chilled PBS gently, centrifuged again at 2,000 × g for 10 min and resuspended in 500 μL fresh pre-chilled PBS to obtain the washed cells. Sialidase activity in the spent growth medium (100 μL) and washed-cell suspension (50 μL) was measured using the MUNANA assay described above, except that fluorescence was monitored continuously in triplicate at 37 °C for 1–2 h.

### 2.6 Growth curve determination for *C. ochracea* strains in TSB

Stationary phase *C. ochracea* cultures were diluted to an OD_600_ of 0.05 in fresh TSB. Growth was monitored in the presence or absence of 200 μM sialidase inhibitor (DANA, siastatin B or oseltamivir) using 200 μL cultures in 96-well microtiter plates. For the growth of the Δ*nanH* mutant and the com-Δ*nanH* complemented strains, the TSB was supplemented with appropriate antibiotics. The OD_600_ was measured every 30 min for 28 h at 37 °C under shaking conditions in CLARIOstar Plus Microplate Reader (BMG Labtech) supplied with 85% N_2_, 10% H_2_, and 5% CO_2_. Growth assays were done in triplicate.

### 2.7 Biofilm quantification

#### 2.7.1 Crystal violet biofilm formation assay

*C. ochracea* WT, Δ*nanH* and comΔ*nanH* strains were anaerobically grown on blood agar for 3 days at 37 °C. Colonies were resuspended in TSB and adjusted to an OD_600_ of 0.278 (~2 × 10^8^ CFU/mL). Then, diluted cultures were added to 96-well flat-bottom polystyrene plates. For examining antibiofilm activities of DANA, siastatin B, or oseltamivir, *C. ochracea* cultures were treated with certain concentrations of the inhibitor (25, 50, 200 and 400 μM). The bacteria without treatment served as the control group. After three days of anaerobic incubation, the biofilms were stained with 80 μL of 0.1% crystal violet (CV) for 15 min, washed with water three times, and then air-dried for 30 min. To quantify biofilm biomass, 200 μL of 33% acetic acid was added to each well and shaken for 1 h. 100 μL of suspension was transferred to a new 96-well polystyrene plate to measure absorbance at 595 nm using a microplate spectrophotometer.

#### 2.7.2 Confocal laser scanning microscopic analysis of biofilms

For Confocal Laser Scanning Microscopy (CLSM), biofilms were grown in *μ*-Slide 8-well chambers with a polymer coverslip (ibidi GmbH, Munich, Germany) by the aforementioned procedures, in the presence or absence of inhibitors. Analogous bacterial cultures without treatment served as the control group. After 72 h, biofilms were stained with SYTO9 and PI by using the Live/Dead BacLightTM viability kit (Thermo Fisher Scientific) for 30 min in the dark at room temperature and the biofilm viabilities were assessed by CLSM equipped with 543 nm HeNe laser and 488 nm Argon laser (Olympus FLUOVIEW FV 1000 with an FV-10 ASW system; Tokyo, Japan). The fluorescence images were analyzed by ImageJ. Each z-stack image was further quantified to determine the average biofilm thickness.

#### 2.7.3 Scanning electron microscopic analysis of biofilms

*C. ochracea* WT, Δ*nanH* and comΔ*nanH* strains were cultured and adjusted as described for the CV biofilm assay. They were incubated in 24-well flat-bottom polystyrene plates with glass coverslips. After 2 h, 6 h, 24 h and 48 h, the medium was removed, and the wells were washed once with PBS. Bacteria attached to the coverslips were fixed overnight at 4 °C with 2% paraformaldehyde and 2.5% glutaraldehyde in PBS. The samples were washed twice with PBS and dehydrated in ethanol (30, 50, 70, 85, 95 and 100%). The samples were then air-dried, coated with platinum, and observed under a scanning electron microscope (SEM; Hitachi Ltd., Tokyo, Japan).

### 2.8 Adhesive and invasion assay

Human gingival epithelial cells (hGECs, CELLnTEC) were cultured in CnT-prime medium with 100 μg/mL Primocin (InvivoGen, San Diego, USA) and placed in the humidified incubator at 37 °C with 5% CO_2_. Adhesion and invasion assays were performed using an adaptation of our previously described method (Li et al., 2025). In brief, *C. ochracea* strains were resuspended in the medium at a MOI of 100 and added to a confluent monolayer of hGECs (∼6×10^5^ cells/well) for incubation at 37 °C for 2 h, respectively. The cells were gently washed with PBS and lysed in DI water for 15 min. Cell lysates were serially diluted and plated on blood agar for CFU counting to determine the total number of *C. ochracea* (adhered or invaded) associated with hGECs. For the invasion assay, the cells were gently washed with PBS and treated with a high-concentration antibiotic mixture (200 μg/mL metronidazole and 300 μg/mL gentamicin) for 1 h to eliminate extracellular bacteria. After the treatment, the cells were washed, lysed, and the cell lysates were diluted and plated as described above. Relative adhesion and invasion were expressed as the total number of bacteria relative to WT, defined as 100%.

### 2.9 Statistical analysis

The data were statistically analyzed by one-way analysis of variance (ANOVA) with Bonferroni’s multiple comparisons test. All of the results were plotted using GraphPad Prism 10 and presented as mean ± SD.

## 3 Results

### 3.1 Screening for exo-α-sialidase in *C. ochracea*

Sialidase activity was detected in *C. ochracea*, consistent with the previous findings (Moncla et al., 1990). This activity was comparable to, albeit weaker than, that of *P. gingivalis* and *T. forsythia* (**Figure 1A**). In the related animal pathogen *C. canimorsus*, the sialidase SiaC was identified as an extracellular lipoprotein anchored in the outer membrane (Mally et al., 2009; Renzi et al., 2015). To localize the enzyme(s) responsible for sialidase activity in *C. ochracea*, we assayed the MUNANA hydrolysis in its spent growth and washed cells, as illustrated in **Figure 1B**. Activity was predominantly cell-associated with weak activity detectable in the spent growth medium, indicating that the enzyme(s) are surface-associated or intracellular (**Figure 1C**). However, their identity remained unknown. Therefore, we performed a BLASTp search using the SiaC gene against the *C. ochracea* CCUG 9716 genome, which identified Coch_0016 (accession number WP_012796827.1), a 515-amino-acid (aa) protein we designated Co-NanH, as a putative sialidase.

Multiple-sequence alignment of *Capnocytophaga* sialidase homologues showed high conservation around the catalytic domain, whereas the non-catalytic domain differs between human- and animal-associated species (**Supplementary Figure 1**). All human *Capnocytophaga* sialidases possess Sec/SPI signal peptides, whereas most animal *Capnocytophaga* sialidases carry Sec/SPII signal peptides (**Supplementary Figure 2**). The Sec/SPII signal of SiaC mediates extracellular localization and supports *C. canimorsus* growth in the presence of murine monocyte–macrophages (Mally et al., 2008). By contrast, Co−NanH features a Sec/SPI signal peptide, consistent with the partial secretion reflected by extracellular activity (**Figure 1C**). To further evaluate its subcellular localization, we conducted additional bioinformatic analyses using PSORTb 3.0 (Yu et al., 2010) and CELLO v2.5 (Yu et al., 2006). Both tools consistently predict Co-NanH to be a periplasmic protein with the potential for membrane association, align with the detection of sialidase activity in membrane-associated fractions (**Figure 1C**). Taken together, these findings indicate that Co−NanH is membrane-associated and capable of secretion.

To further characterize Co-NanH, phylogenetic analysis of sialidases from species including *Capnocytophaga*, representative oral periodontal pathogens (*e.g.*, *P. gingivalis*, *T. denticola* and *T. forsythia*), and *Bacteroides fragilis* revealed that human- and animal-associated *Capnocytophaga* sialidases form separate subclusters within a *Capnocytophaga* clade that is distinct from the red-complex sialidases. *Capnocytophaga* sialidases are most closely related to Tf-NanH and *B. fragilis* sialidase homologue (**Figure 1D**).

### 3.2 Domain structure of Co-NanH

**Figure 2:**
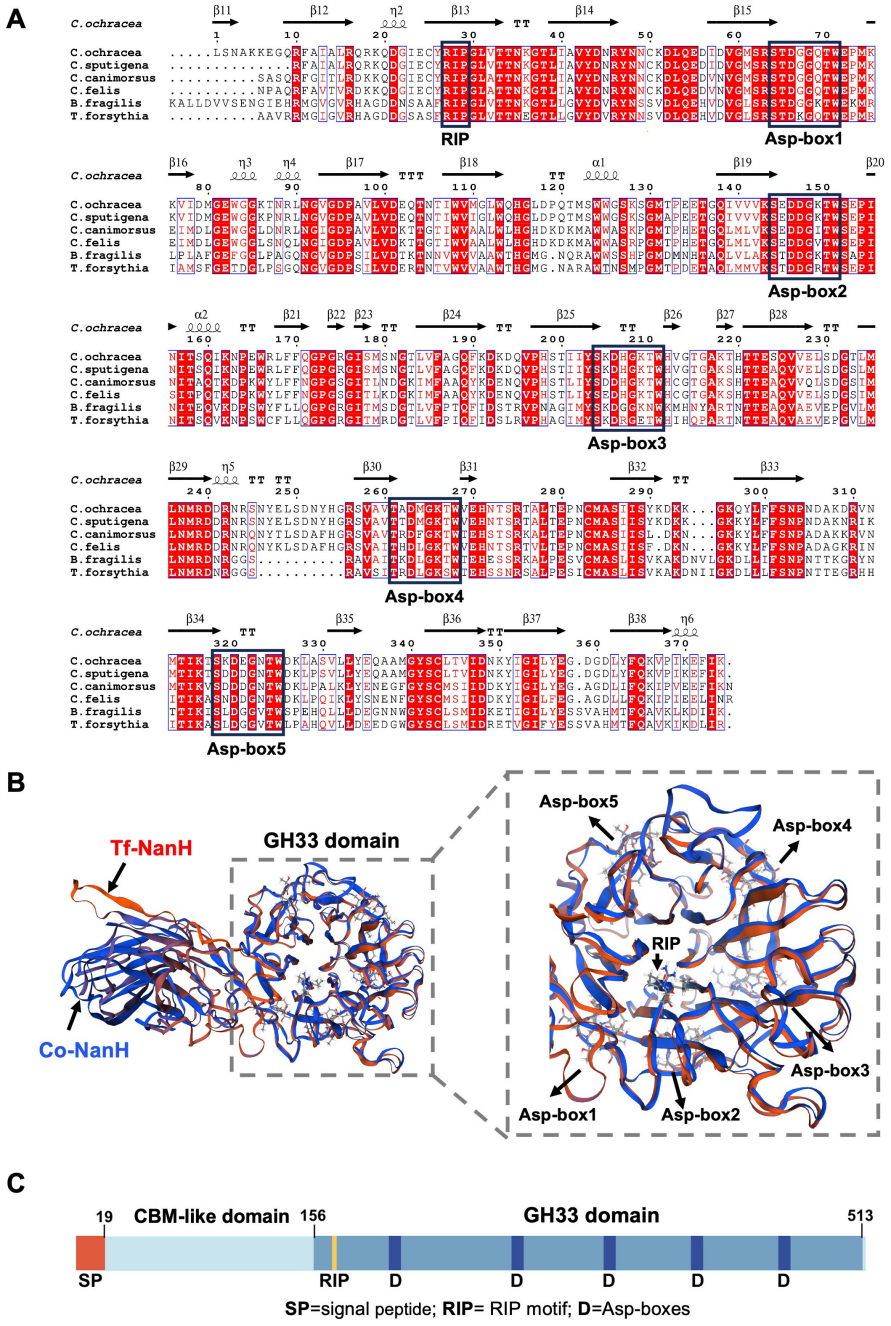
Domain and sequence analysis, and a putative 3D model of Co-NanH. **(A)** Multiple sequence alignment of the respective catalytic domains of sialidases from diverse organisms: *C. ochracea* CCUG 9716 (WP_012796827.1), *C. sputigena* CCUG 9714 (WP_002681834.1), *C. canimorsus* Cc5 (WP_013996593.1), *C. felis* DSM 10725 (WP_227977333.1), *B. fragilis* NCTC 9343 (WP_005808991.1) and *T. forsythia* 92A.2 (WP_014225510.1). The ‘RIP’ motif and five respective ‘Asp-box’ are indicated with the box. This panel was prepared using ESPript 3.0 (Robert and Gouet, 2014). **(B)** Structural comparison of Co-NanH (blue) with Tf-NanH (red, PDB code: 7QYJ) by SwissModel comparisons. **(C)** Schematic diagram showing the predicted domain structure and conserved motifs present within Co-NanH. Features include the signal peptide (SP, red), CBM-like domain (light blue), the GH33 domain (blue), the RIP motif (RIP, yellow), and the Asp-box (D, dark blue). The figure was prepared using IBS 1.0 (Liu et al., 2015).

Sequence alignment revealed that the catalytic domain of Co-NanH possesses the conserved ‘RIP’ motif and five ‘Asp-box’ repeats (S-X-D-X-G-X-T-W/F), canonical features of bacterial sialidases. The catalytic domain shares high levels of sequence similarity to those of *T. forsythia* and *B. fragilis* (**Figure 2A**). Structural modelling with SwissModel (Waterhouse et al., 2018) indicated that the GH33 domain structure of Co-NanH (blue) closely overlaps with that of Tf-NanH (red), sharing ‘RIP’ motif and ‘Asp-box’ repeats (**Figure 2B**). Tf-NanH possesses an N-terminal carbohydrate-binding module (CBM) in addition to its catalytic domain (Shoseyov et al., 2006; Frey et al., 2018; Satur et al., 2022). Similarly, Co-NanH is predicted to contain an N-terminal Sec/SPI signal peptide (residues 1-19), a CBM-like domain (residues 20-155), and a C-terminal GH33 catalytic domain (residues 156-513) (**Figure 2C**). Notably, their N-terminal non-catalytic domains exhibit substantial variation (**Supplementary Figure 3**).

Analysis of the genomic context highlighted a critical distinction between Co-NanH and Tf-NanH. Specifically, Tf-NanH is immediately adjacent to genes encoding proteins involved in sialic acid uptake and catabolism (Roy et al., 2010), a characteristic absent from the *C. ochracea* sialidase locus associated with Co-NanH. However, gene synteny at the sialidase locus is highly conserved across the *Capnocytophaga* genome but shows subtle distinctions between human and animal-associated species through the FlaGs webserver (WebFlaGs) (**Supplementary Figure 4**).

### 3.3 Biophysical and biochemical characterization of recombinant Co-NanH

To better understand the biophysical and biochemical properties of Co-NanH, we cloned the coding sequence, lacking the signal peptide, into pET28a (**Supplementary Figure 5A**). SDS-PAGE and Coomassie brilliant blue staining of IPTG-induced cultures of DH5aCo-NanH did not clearly reveal overexpression of the expected ~58 kDa protein (**Supplementary Figure 5B**). Purified recombinant Co-NanH (molecular weight [MW], 59 kDa) migrated at *ca.* 58 kDa upon size-exclusion chromatography (SEC) analysis, consistent with a stable monomer in solution (**Supplementary Figure 5C**).

**Figure 3:**
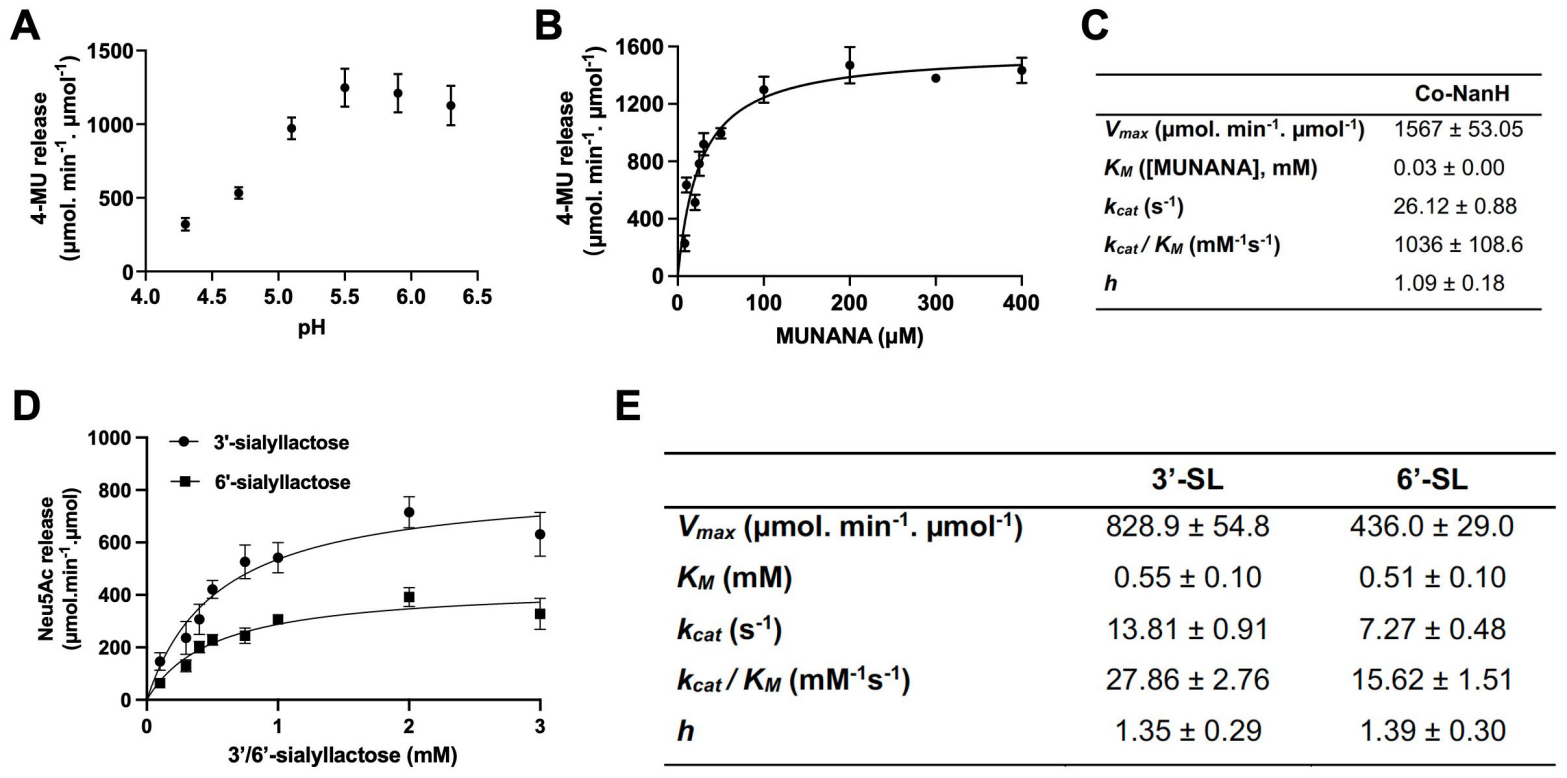
Sialidase activities of Co-NanH against MUNANA, 3’-SL and 6’-SL. **(A)** MUNANA was incubated with Co-NanH, and the sialidase activities were monitored continuously in buffers with various pH values (4.3, 4.7, 5.1, 5.5, 5.9, and 6.3). **(B)** Variable concentrations of MUNANA were incubated with Co-NanH at the optimum pH. The *y*-axes show the reaction velocity in units of micromoles of 4-MU by-product formed per minute per micromole of Co-NanH. The *x*-axes show MUNANA concentration in micromole units. **(C)**. Table summarizing the kinetic parameters obtained: maximum reaction velocity (*V*_max_), *K_M_* for MUNANA in millimolar units, *k_cat_* in units of 4-MU molecules formed per second, and *k_cat_*/*K_M_*. All reactions were performed in triplicate, reporting the means ± standard deviation. **(D)** Various concentrations of 3’-SL and 6’-SL were incubated with Co-NanH. The rate of sialic acid (Neu5Ac) release was determined by comparison with a previously generated standard curve of Neu5Ac over the same concentration range. Michaelis–Menten plot, rate of Neu5Ac release (V0, Neu5Ac release μmol.min^−1^ μmol^−1^), plotted against [3’/6’-sialyllactose] (mM) using Prism 10 (GraphPad). Error bars, sem. **(E)** Table summarizing the kinetic parameters obtained: *V*_max_*, K_M_* for 3’-SL/6’-SL in millimolar units, *k_cat_* in units of Neu5Ac molecules formed per second and *k_cat_*/*K_M_*.

Co-NanH displayed the optimum activity at *ca.* pH 5.5, with activity declining at higher pH values (**Figure 3A**). This pH profile aligns with the subgingival biofilm niche of *C. ochracea*, where alkaline GCF is associated with periodontitis progression (Bickel and Cimasoni, 1985; Khurshid et al., 2017; Frey et al., 2018). Kinetic analysis with MUNANA yielded a *K_M_* of 26.0 ± 3.11 μM and a *k_cat_* of 26.12 ± 0.88 s^-1^ under the tested conditions, resulting in a catalytic efficiency (*k_cat_/K_M_*) comparable to that of Tf-NanH (2.1 μM/s) (Frey et al., 2018) (**Figures 3B, C**).

To assess substrate specificity, we tested activity against host-like glycans using the TBA assay as previously described (Roy et al., 2011). Co-NanH cleaved both α2,3- and α2,6-linked sialic acid from sialyllactose, with slightly higher affinity (lower *K_M_*) for 6’-SL (*K_M_* = 0.51 ± 0.10 mM) than 3’-SL (*K_M_* = 0.55 ± 0.10 mM). However, catalytic efficiency was nearly twofold greater for 3’-SL (*k_cat_/K_M_* = 27.86 ± 2.76 mM/s) than for 6’-SL (*k_cat_/K_M_* = 15.62 ± 1.51 mM/s) (**Figures 3D, E**).

### 3.4 Susceptibility of Co-NanH to DANA, siastatin B, and oseltamivir

**Figure 4:**
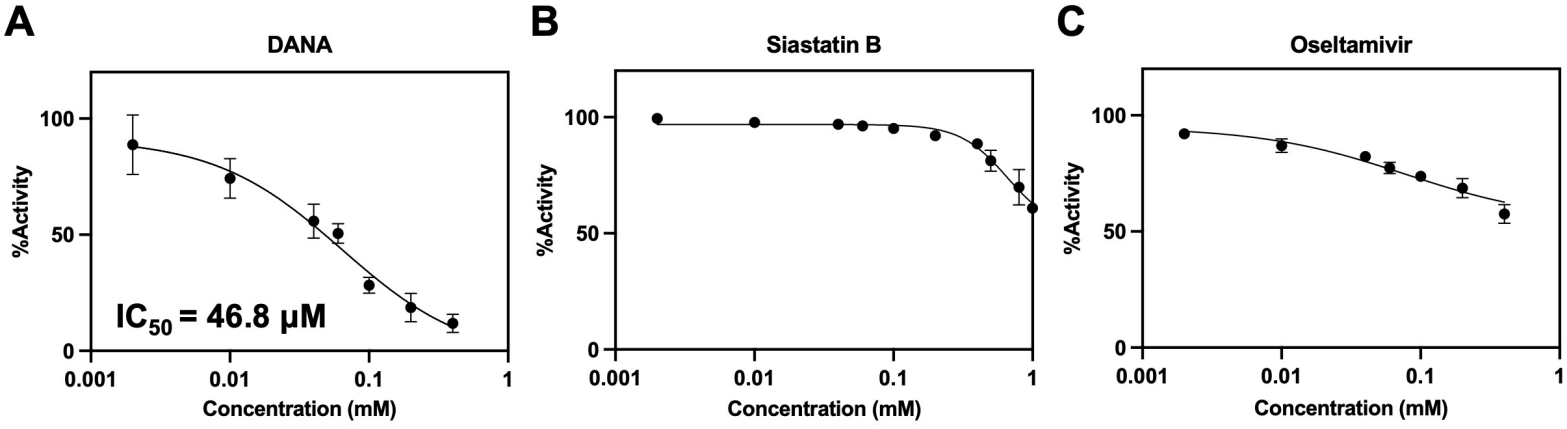
Inhibition effect of DANA, siastatin B and oseltamivir on the sialidase activities of Co-NanH. The inhibitory effects of **(A)** DANA, **(B)** siastatin B and **(C)** oseltamivir on Co-NanH sialidase activity were performed using MUNANA-based assays. The data shown here are the mean ± SD from three independent experiments.

Given the reported varying efficacy of sialidase inhibitors against bacterial enzymes (Keil et al., 2022), we investigated the inhibition profile of Co-NanH. We assessed three well-characterized sialidase inhibitors via MUNANA assay: DANA, a transition-state sialic acid analogue; siastatin B, a natural product inhibitor; and oseltamivir, an antiviral sialidase inhibitor. While all three compounds reduced Co-NanH activity, their potencies differed markedly. DANA proved to be a potent inhibitor, yielding an IC_50_ value of approximately 46.8 μM. In contrast, siastatin B and oseltamivir demonstrated only modest inhibition under identical experimental conditions (**Figures 4A–C**).

### 3.5 Effects of inhibitors on *C. ochracea* growth and biofilm formation

**Figure 5:**
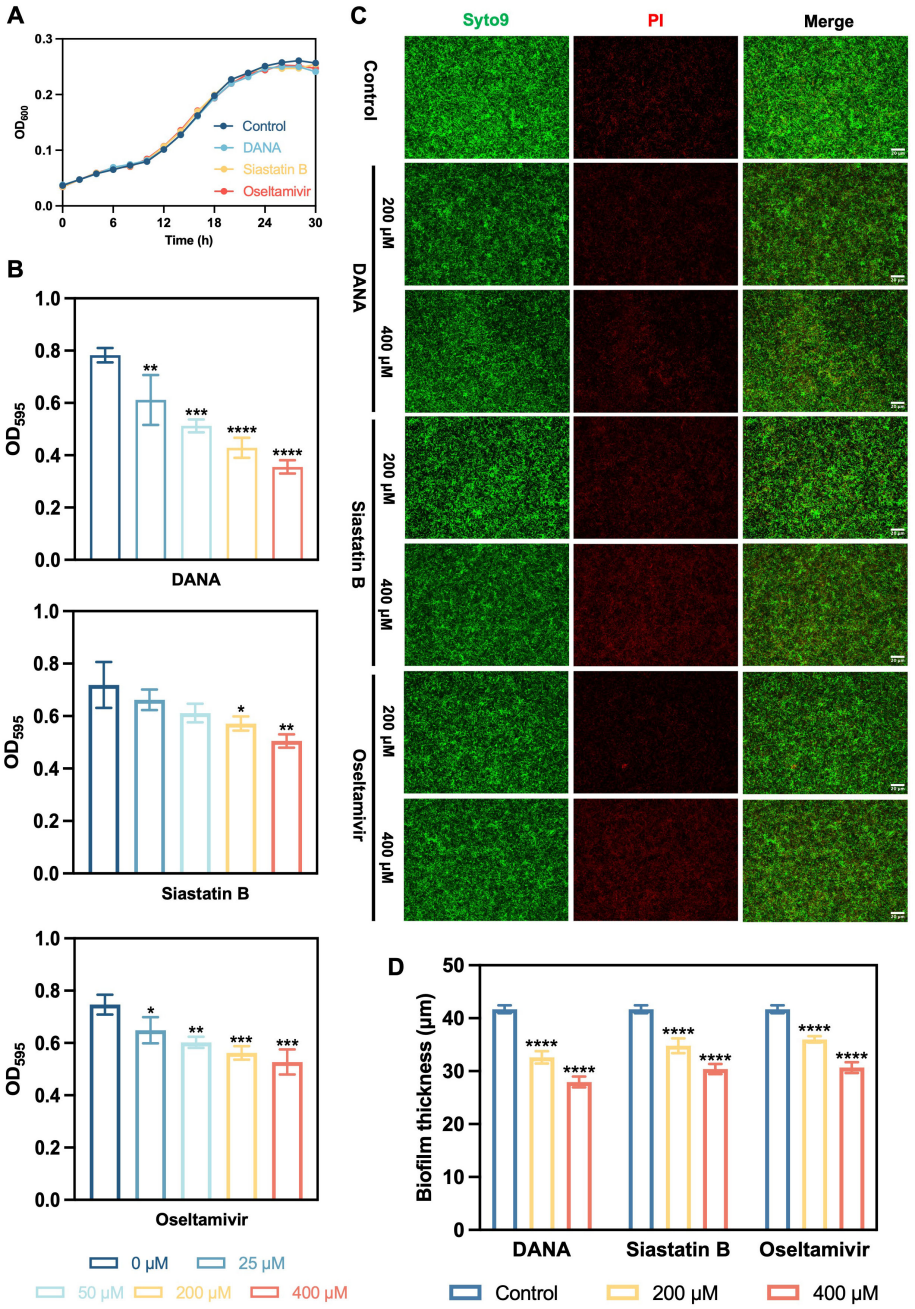
Inhibition effect of DANA, siastatin B and oseltamivir on the biofilm formation of *C. ochracea.* **(A)** Growth curve of *C. ochracea* in TSB with or without 200 μM DANA, siastatin B or oseltamivir. **(B)** *C. ochracea* was cultured in the absence or the presence of DANA, siastatin B or oseltamivir with certain concentrations (25, 50, 200 and 400 μM). After 72 h, biofilms were stained with crystal violet and the optical density at 595 nm was determined. **(C)** Biofilms were grown in the absence or the presence of 200 μM or 400 μM DANA, siastatin B or oseltamivir, respectively. After 72 h, biofilms were stained with SYTO9 (green) and propidium iodide (PI, red) and imaged using the Olympus confocal microscopy system at ×60. ImageJ was used to determine from z-stacks. Scale bars represent 20 μm. **(D)** Biofilm thickness (μm) was determined from z-stacks. The data shown here are the mean ± SD from three independent experiments. *p < 0.05, **p < 0.01, ***p < 0.001, ****p < 0.0001 as determined by one-way ANOVA. * Indicated the comparison between the control group and the individual-treated group.

Given the significance of various bacterial sialidases in diseases and their feasibility as potential therapeutic targets, sialidase inhibitors have also been explored as antibacterial agents (Govinden et al., 2018; Wang, 2020; Yu et al., 2021). Growth assays showed that treatment of *C. ochracea* with 200 μM DANA, siastatin B or oseltamivir did not show an obvious planktonic growth defect (**Figure 5A**). By contrast, biofilm assays revealed that all three inhibitors at ≥ 200 μM efficiently reduced biofilm biomass, as measured by crystal violet staining (**Figure 5B**). Confocal imaging confirmed that biofilms treated with inhibitors exhibited reduced thickness and bacterial density, with markedly weaker fluorescence signals compared to controls (**Figures 5C, D**).

### 3.6 Deletion of nanH impairs biofilm formation and initial attachment

To test the hypothesis that sialidase activity contributes to biofilm formation, we constructed a *nanH* gene deletion mutant (Δ*nanH*) via allelic exchange mutagenesis (**Supplementary Figure 6A**). PCR analysis confirmed replacement of *nanH* with an erythromycin-resistant cassette (Erm^r^) (**Supplementary Figure 6B**). A complemented strain (comΔ*nanH*) was generated by inserting full length *nanH* into the Tc^r^ cassette on the chromosome of the mutant.

**Figure 6:**
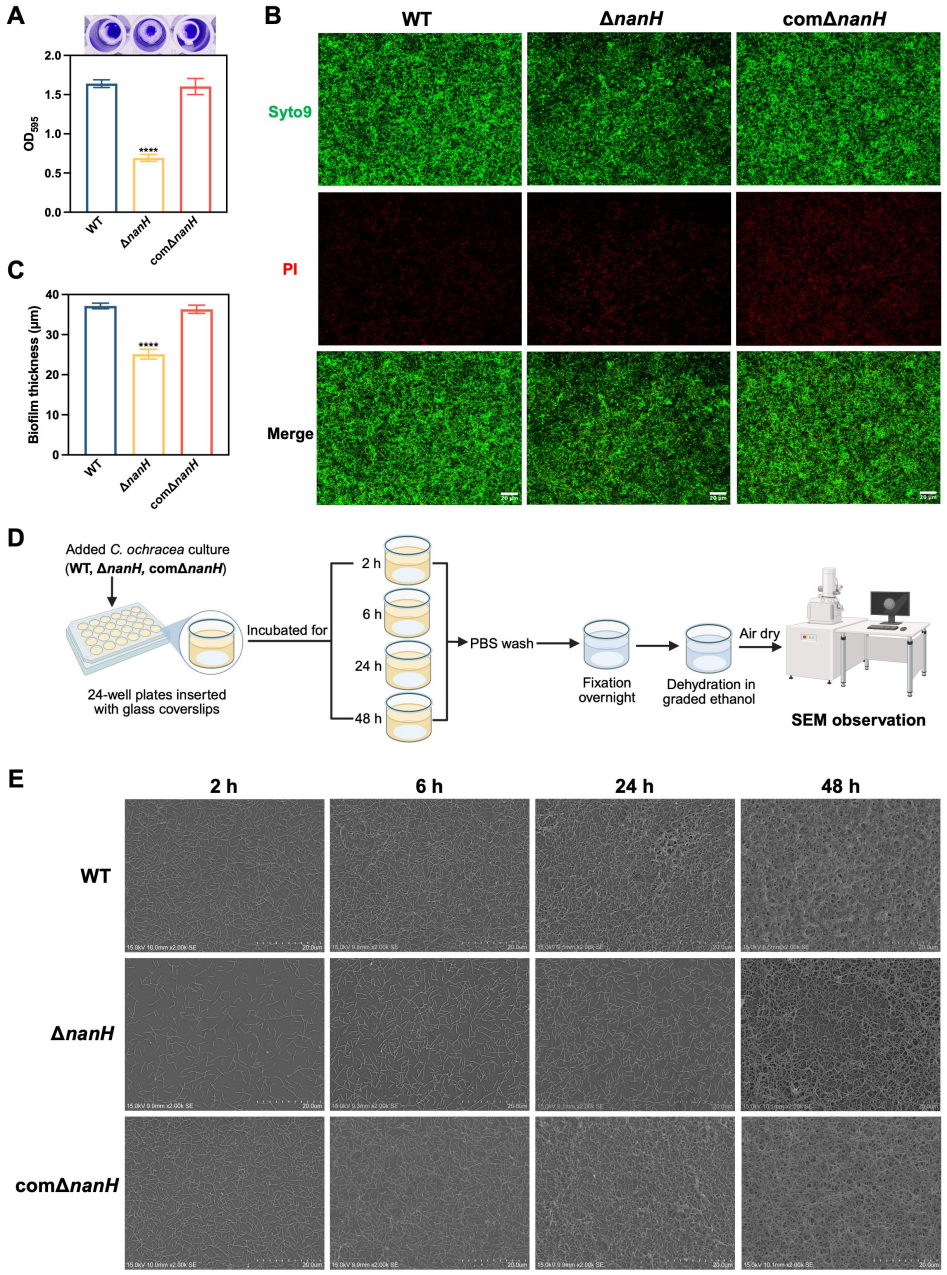
Biofilm formation by *C. ochracea*, sialidase-deficient Δ*nanH*, and *nanH* complemented strain comΔ*nanH*. **(A)** *C. ochracea* WT, Δ*nanH* and comΔ*nanH* strains were cultured in the 96- well plate. After 72 h, biofilms were stained with crystal violet and the optical density at 595 nm was determined. Representative images of crystal violet staining are shown above the graph. **(B)** *C. ochracea* WT, Δ*nanH* and comΔ*nanH* strains were cultured in the ibidi *μ*-Slide 8-well chambers. After 72 h, biofilms were stained with SYTO9 (green) and PI (red) and imaged using the Olympus confocal microscopy system at ×60. ImageJ was used to determine from z-stacks. Scale bars represent 20 μm. **(C)** Biofilm thickness (μm) was determined from z-stacks. The data shown here are the mean ± SD from three independent experiments. ****p < 0.0001 as determined by one-way ANOVA. * Indicated the comparison between the control group and the individual-treated group. **(D)** Schematic diagram showing the workflow for comparing the attachment ability among *C. ochracea* WT, Δ*nanH* and comΔ*nanH* strain through SEM observation. **(E)** SEM images of *C. ochracea* WT, Δ*nanH* and comΔ*nanH* attached to the coverslips for 2 h, 6 h, 24 h and 48 h (scale bar: 20 μm). Part of the scheme was created in BioRender. See **Supplementary Materials** and Methods for detailed experimental protocols of *C. ochracea* Δ*nanH* and comΔ*nanH* strains construction.

The Δ*nanH* mutant exhibited planktonic growth comparable to WT and comΔ*nanH* in the nutrient-rich medium utilized for biofilm formation (**Supplementary Figure 7**), confirming *nanH* is dispensable for growth. Crucially, while whole-cell sialidase activity was detected in WT and comΔ*nanH* strains, it was completely absent in Δ*nanH* (**Supplementary Figure 8**), indicating that *nanH* encodes the primary and likely sole sialidase responsible for extracellular and cell-associated activities. Biofilm formation by Δ*nanH* was significantly reduced, approximately 3-fold versus WT, evidenced by decreased crystal violet staining (**Figure 6A**), thinner pellicles, reduced surface coverage, and diminished biofilm thickness (WT: 37.1 μm, Δ*nanH*: 25.1 μm, comΔ*nanH*: 36.3 μm) (**Figures 6B, C**). Furthermore, scanning electron microscopy analysis of 48-hour biofilms (**Figure 6D**) revealed that the defect stemmed from impaired initial bacterial attachment in Δ*nanH* during early biofilm development (**Figure 6E**).

### 3.7 *nanH* is involved in the adhesion and invasion of oral epithelial cells

**Figure 7:**
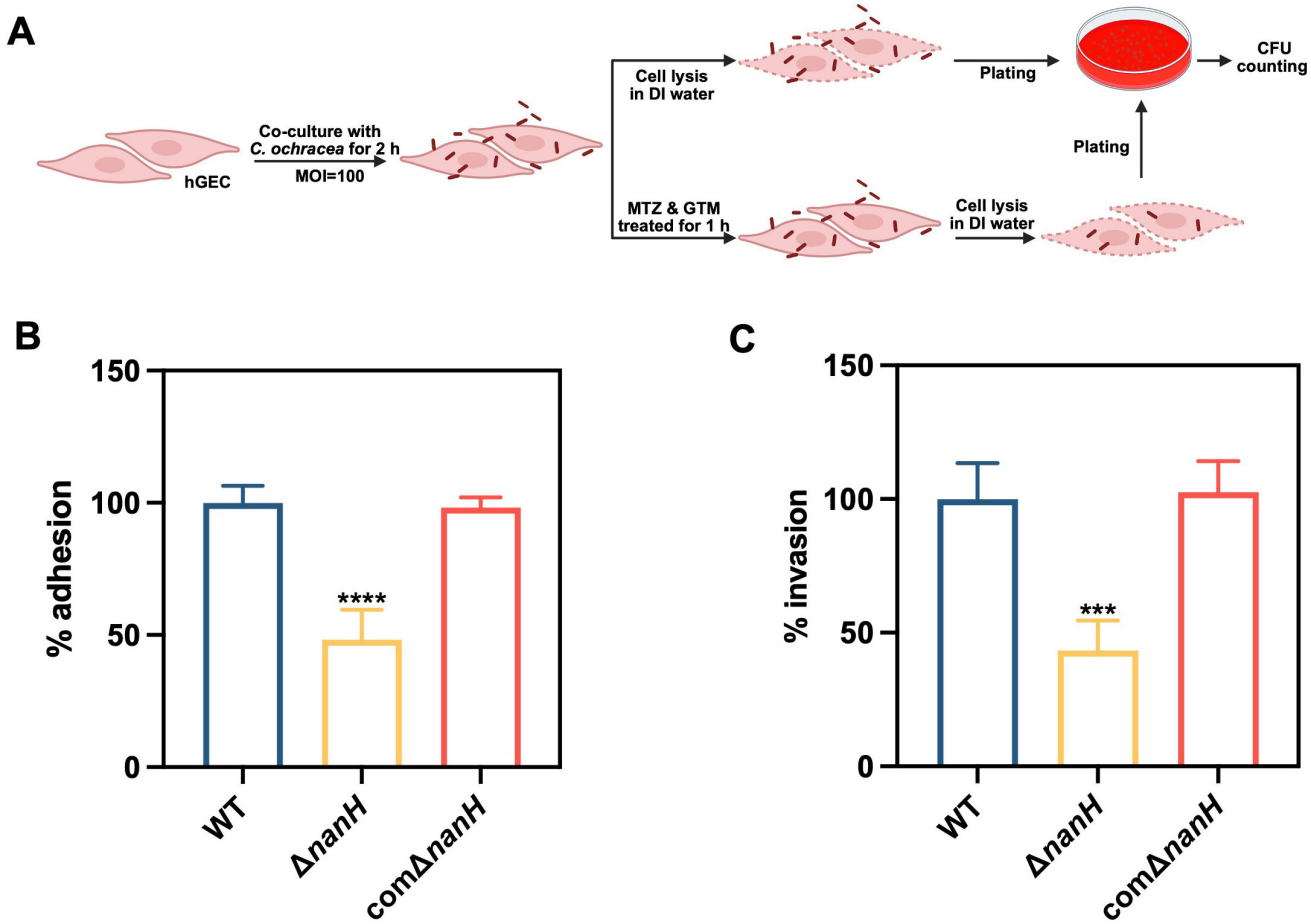
Adhesion and invasion of *C. ochracea* strains to epithelial cell lines. **(A)** Illustration of the *C. ochracea* adhesion and invasion into hGECs. Antibiotic protection assays were performed. Part of the scheme was created in BioRender. The **(B)** adhesion and **(C)** invasion levels of the indicated *C. ochracea* strains to hGECs are expressed as the total number of bacteria relative to WT, defined as 100%. The data shown here are the mean ± SD from three independent experiments. ***p < 0.001, ****p < 0.0001 as determined by one-way ANOVA.

Given that epithelial cells are decorated with a variety of sialic acid-containing glycoconjugates, which may facilitate bacterial attachment or provide bacteria with a carbon and nitrogen source for nutrition, we assessed *nanH*’s role in host interactions. Using hGECs at an MOI of 1:100, typical of invasion rates commonly observed for oral anaerobes such as *P. gingivalis*, *T. forsythia*, and *Fusobacterium spp (*Naylor et al., 2017). We evaluated bacterial adhesion and invasion (**Figure 7A**). Δ*nanH* showed significantly reduced attachment to hGECs compared to WT and comΔ*nanH* (**Figure 7B**). Similarly, invasion assays revealed that the Δ*nanH* mutant exhibited impaired internalization (**Figure 7C**), confirming that *nanH* mediates adhesion to and internalization by gingival epithelial cells.

## 4 Discussion

Periodontal diseases arise from a dysbiotic subgingival microbiota and a corresponding dysregulated host immune response. Members of the ‘red complex’ are central to this process, colonizing periodontal pockets and forming biofilms that protect pathogens and enhance their persistence. Within these biofilms, bacterial sialidases modify host glycoproteins, thereby promoting bacterial adhesion and immune evasion. This interaction between periodontal pathogens, biofilms, and sialidase activity significantly contributes to periodontal progression. This study provides the first comprehensive biochemical and functional characterization of the sialidase from *C. ochracea*, an opportunistic periodontal pathogen. Our findings demonstrate that Co-NanH shares significant structural enzymatic homology with sialidases from the ‘red complex’ pathogens, particularly *T. forsythia*, and plays a crucial role in biofilm formation and host cell interactions.

It is noteworthy that all red complex bacteria harbor one or two sialidases, which share conserved biochemical features. Co-NanH exhibits features conserved among them. Like Tf-NanH, Co-NanH is a dual-domain protein featuring an N-terminal CBM-like domain and a C-terminal catalytic domain containing the canonical ‘RIP’ motif and ‘Asp-box’ repeats (Frey et al., 2018; Satur et al., 2022). Both enzymes possess a signal peptide for secretion and are active over a broad acidic pH range, optimal at pH 5.5 (Frey et al., 2018). Sialidase activity to remain active across a wide range of pH conditions enhances the capacity of periodontal bacteria to colonize subgingival plaque biofilms. This observation further supports evidence of their heightened metabolic activity within the periodontal pocket. In addition, they efficiently cleave both α2,3- and α2,6-linked sialic acids of glycoproteins (Frey et al., 2018) (**Figure 3D**). Moreover, their comparable activity against the synthetic substrate MUNANA and similar susceptibility to the inhibitor DANA further underscore their functional homology (Frey et al., 2018; Satur et al., 2022) (**Figures 3B** and **4A**).

Despite these similarities, a key structural difference exists. SEC analysis indicates Co-NanH is a stable monomer (**Supplementary Figure 5C**), whereas Tf-NanH exists as a dimer (Satur et al., 2022). This distinction, along with minor variations in the N-terminal domain, suggests subtle functional adaptation between two enzymes. The CBM domain in Tf-NanH was proven to be a key ligand for host-pathogen interactions during cellular invasion (Frey et al., 2018). The presence of a CBM-like domain in Co-NanH, homologous to that in *T. forsythia* and *B. fragilis* sialidase, suggests a conserved function in host-pathogen interactions (Thompson et al., 2009). Moreover, the *C. ochracea* genome notably lacks the sialic acid transport and utilization locus found in *T. forsythia (*Roy et al., 2010) (**Supplementary Figure 4**). This suggests that *C. ochracea* may not directly metabolize the sialic acid it cleaves. Instead, Co-NanH could function in a cross-feeding mechanism within the subgingival biofilm community. A similar mutually beneficial relationship was proven to exist between *Gardnerella vaginalis* and *Fusobacterium nucleatum*, facilitating their colonization and contributing to vaginal dysbiosis (Agarwal et al., 2020). *C. ochracea* is known to coaggregate with *F. nucleatum*, a critical bridging organism in dental plaque (Weiss et al., 1987; Kolenbrander et al., 2010; Okuda et al., 2012). We hypothesize that Co-NanH desialylates host glycoproteins, exposing penultimate galactose residues that serve as binding sites for *F. nucleatum*. The liberated sialic acid could then be consumed as a nutrient by *F. nucleatum* or other neighboring species. Such inter-species foraging, enabled by sialidase activity, likely promotes the maturation and stability of the dysbiotic biofilm, contributing to disease progression (Robinson et al., 2017).

Functionally, our results establish Co-NanH as a key factor in *C. ochracea* colonization. Genetic inactivation of *nanH*, the sole sialidase-encoding gene identified in the genome, resulted in a significant reduction in biofilm formation (**Figure 6**). This phenotype parallels findings in other periodontal pathogens, such as *P. gingivalis* and *T. forsythia*, in which sialidase deletion also impairs biofilm development. For example, the Δ*PG0352* mutant formed significantly less biofilm (Li et al., 2012), and Tf-NanH is essential for biofilm formation on glycoprotein-coated surfaces (Roy et al., 2011). While the precise molecular mechanism driving the biofilm defect in *C. ochracea* Δ*nanH* mutant remains to be elucidated, our data indicate a marked reduction in initial bacterial adhesion (**Figure 6E**). This suggests that, beyond its enzymatic activity, Co-NanH may function directly as an adhesin, facilitating the early stages of biofilm architecture. Given that *C. ochracea* is an early colonizer, its ability to adhere to both hard tooth surfaces and mucosal soft surfaces is pivotal for the succession of dental biofilms. Mechanistically, sialic acid residues often cap the host glycans, masking underlying bacterial binding sites. The cleavage of these terminal residues by sialidases exposes cryptic receptors, such as galactose (Engibarov et al., 2025), which serve as high-affinity ligands for adhesins of both the producer and secondary colonizers. By acting as a ‘trigger’ at the initial stage of colonization, Co-NanH likely facilitates the assembly of robust polymicrobial communities. Alongside previously identified factors like gliding motility and *LuxS (*Hosohama-Saito et al., 2016; Kita et al., 2016), sialidase represents a new and important contributor to intra- and interspecies biofilm formation in *C. ochracea*, underscoring the potential role of these colonization-associated factors in the pathogenesis of biofilm-related diseases.

Beyond its role in biofilm architecture, our findings suggest that Co-NanH significantly contributes to the interaction of *C. ochracea* with host epithelial cells. Bacterial sialidases are well-documented virulence factors that enhance pathogenicity by removing terminal sialic acids from host glycoconjugates, unmasking hidden epitopes that enhance bacterial adhesion and invasion (Corfield, 1992; Kim et al., 2011; Sudhakara et al., 2019). This enzymatic ‘unmasking’ is particularly relevant for periodontal pathogens, as oral epithelial cells are heavily decorated with sialylated receptors that can obscure bacterial binding sites (Severi et al., 2007; Honma et al., 2011). In the current study, the Δ*nanH* mutant exhibited markedly attenuated adhesion to and internalization by oral epithelial cells compared to the WT and complemented strains (**Figure 7**). This aligns with the established role of sialidase in pathogenesis, where enzymatic activity unmask cryptic host cell receptors to facilitate bacterial attachment and entry (Sudhakara et al., 2019; Chen et al., 2024). Analogous mechanisms have been described for *T. forsythia*, where Tf-NanH is essential for unmasking epithelial binding sites (Honma et al., 2011), and for *P. gingivalis*, where sialidase activity inhibition with zanamivir significantly reduces host cell attachment (Frey et al., 2019). Our data strongly suggest that Co-NanH performs a similar function for *C. ochracea*, positioning this organism as an accessory pathogen that contributes to the virulence of the overall microbial community.

Finally, we observed functional divergence between human- and animal-associated *Capnocytophaga* species. Unlike the lipoprotein SiaC of *C. canimorsus*, which is tethered to the outer membrane via a Sec/SPII signal peptide (Mally et al., 2008) (**Supplementary Figure 2**), representative Co-NanH possesses a standard Sec/SPI signal peptide and is found in both secreted and cell-associated fractions (**Figure 1C**). We speculate that the N-terminal domain of Co-NanH may mediate its non-covalent anchoring to the cell surface, allowing it to function both locally and distally.

## 5 Conclusion

In summary, this study identifies the *C. ochracea* sialidase, Co-NanH, as a multifunctional virulence factor. It shares core biochemical attributes with sialidases from cornerstone periodontal pathogens but also possesses unique structural and regulatory features. Our findings demonstrate its critical roles in biofilm formation and host cell interaction, providing a mechanistic basis for the opportunistic pathogenicity of *C. ochracea*. These results highlight how colonization-associated factors from a commensal organism can contribute to the dysbiotic shifts that drive periodontal disease. Future work should focus on elucidating the molecular mechanisms by which Co-NanH modulates biofilm architecture and on investigating its clinical prevalence and activity in states of periodontal health and disease.

## Data Availability

The raw data supporting the conclusions of this article will be made available by the authors, without undue reservation.
