# Supplementary material for: Capnocytophaga ochracea sialidase drives its biofilm maturation and host cell interactions
Source: Front Cell Infect Microbiol. 2026 Jun 19;16:1841812. doi: 10.3389/fcimb.2026.1841812 (PMC13328365; doi:10.3389/fcimb.2026.1841812)
Supplement: Supplementary file 1 [file DataSheet1.docx]

**Supplementary materials**

***Capnocytophaga ochracea* sialidase drives its biofilm maturation and host cell interactions**

**Jing He^1,2,3,4^, Shouliang Zhao^5*^, Yanqin Ju^5*^**

^1^Department of Endodontics, Shanghai Ninth People’s Hospital, Shanghai Jiao Tong University School of Medicine, Shanghai, China; ^2^College of Stomatology, Shanghai Jiao Tong University, Shanghai, China; ^3^National Clinical Research Center for Oral Diseases, National Center for Stomatology, Shanghai, China; ^4^Shanghai Key Laboratory of Stomatology, Shanghai, China; ^5^Department of Stomatology, Huashan Hospital, Fudan University, Shanghai 200040, P. R. China

**Supplementary Figures**

**
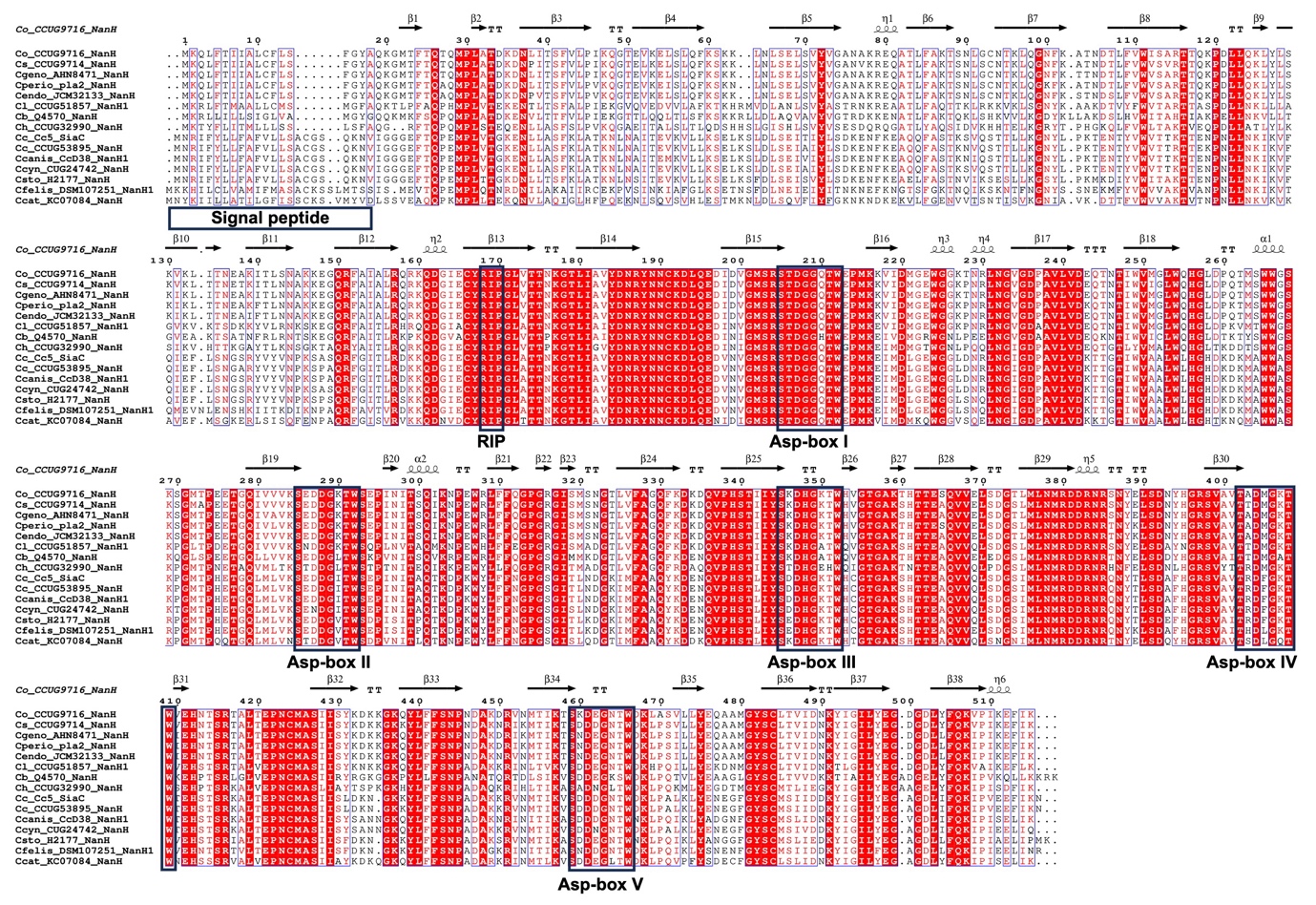
**

**Fig. S1. Multiple sequence alignment of the amino acid sequences of sialidase homologues from representative strains in the *Capnocytophaga* genus.** Multiple sequence alignment of the respective sialidase homologues in the *Capnocytophaga* genus. The signal peptides, RIP motifs and five Asp-boxes are indicated with brackets. Abbreviations: *C. ochracea* CCUG 9716 (Co_CCUG9716_NanH, 515 aa, WP_012796827.1); *C. sputigena* CCUG 9714 (Cs_CCUG9714_NanH, 515 aa, WP_002681834.1); *C. genosp*. AHN8471 (Cgeno_AHN8471_NanH, 515 aa, WP_203093975.1); *C. periodontitidis* pla2 (Cp_pla2_NanH, 522 aa, WP_198475712.1); *C. endodontalis* JCM32133 (Cendo_JCM32133_NanH 515 aa, WP_088594018.1); *C. leadbetteri* CCUG 51857 (Cl_CCUG51857_NanH1, 517 aa, WP_107782516.1); *C. bilenii* Marseille-Q4570 (Cb_Q4570_NanH, 515 aa, WP_208059185.1); *C. haemolytica* CCUG 32990 (Ch_CCUG32990_NanH, 516 aa, WP_066428668.1); *C. canimorsus* Cc5 (Cc_Cc5_SiaC, 522 aa, WP_013996593.1); *C. canimorsus* CCUG 53895 (Cc_CCUG53895_NanH, 522 aa, WP_042000364.1); *C. canis* CcD38 (Ccanis_CcD38_NanH1, 522 aa, WP_042345037.1); *C. cynodegmi* CCUG 24742 (Ccyn_CCUG24742_NanH, 522 aa, WP_026193862.1); *C. stomatis* H227 (Csto_H227_NanH, 523 aa, WP_095895319.1); *C. felis* DSM 10725 (Cfelis_DSM10725_NanH1, 525 aa, WP_227977333.1); *C. catalasegens* KC07084 (Ccat_KC07084_NanH, 523 aa, WP_264846867.1). The figure was prepared using ESPript 3.0.

**
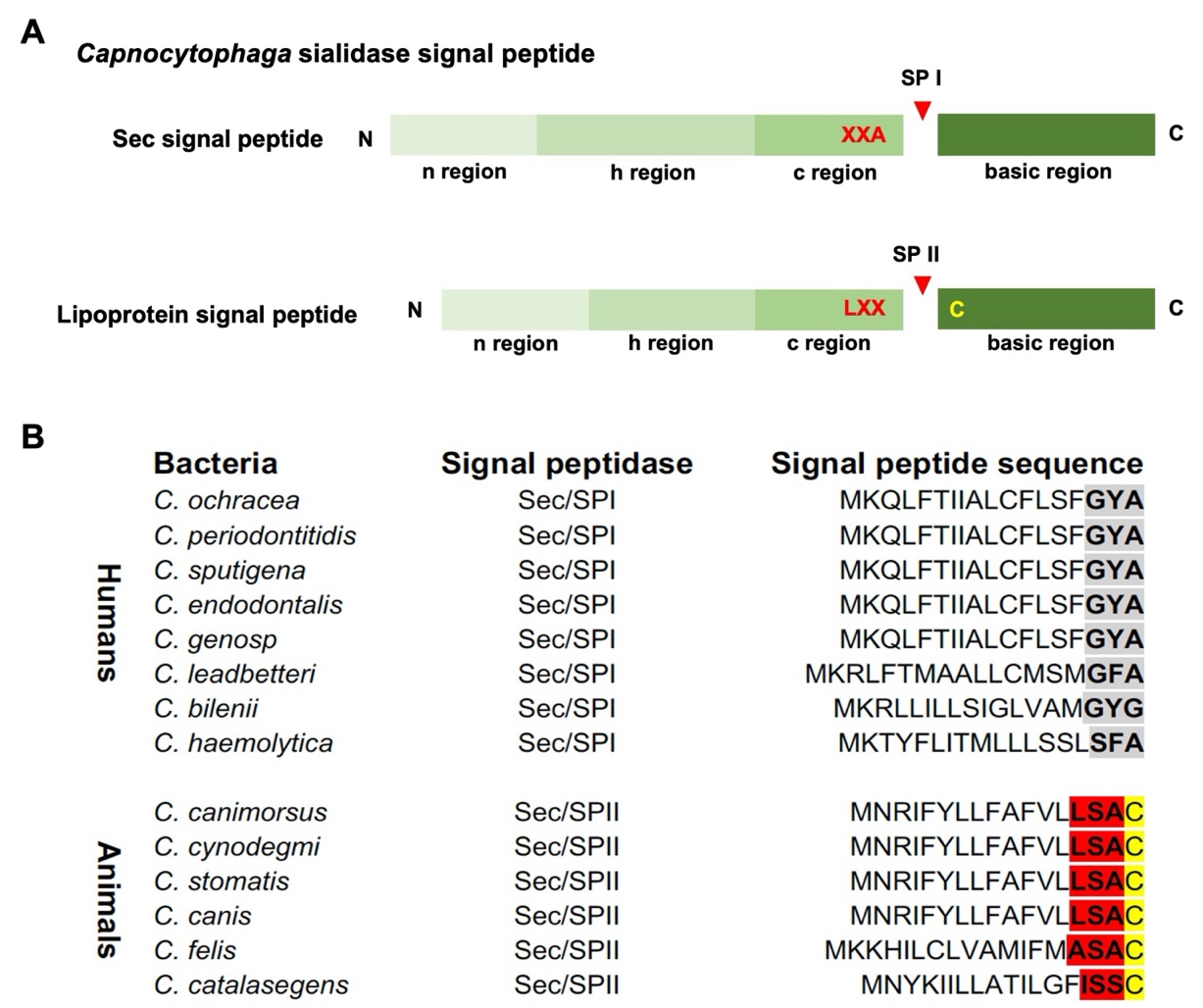
**

**Fig. S2. Sialidase signal peptides encoded by human and animal *Capnocytophaga* species.** (**A**) Schematic representations of the Sec-type signal peptide and the lipoprotein signal peptide, differ in transport pathways and the cleavage characteristics of SPase. The various regions (n, h, c, and basic region) of the signal peptides are indicated. A consensus motif exists before the cleavage site in the C-region (XXA: Ala/Ser/Leu/Ile/Val-X-Ala; LXXC: Leu-Ala/Ser-Gly/Ala-Cys). The SP cleavage site is represented with a red arrow. N and C indicate the amino and carboxyl-terminus, respectively. SPI: Type I signal peptidase; SPII: Type II signal peptidase. (**B**) Table summarizing the signal peptides in sialidases from *Capnocytophaga* species, with features of signal peptidase and the amino acid sequence of the signal peptide. The residues related to the predicted SPase I or SPase II cleavage sites are indicated with bold text and are shaded grey and red, respectively. For lipoprotein signal peptides, the conserved cysteine residue, which is part of the lipobox motif and resides in the basic region, is shaded yellow.

**
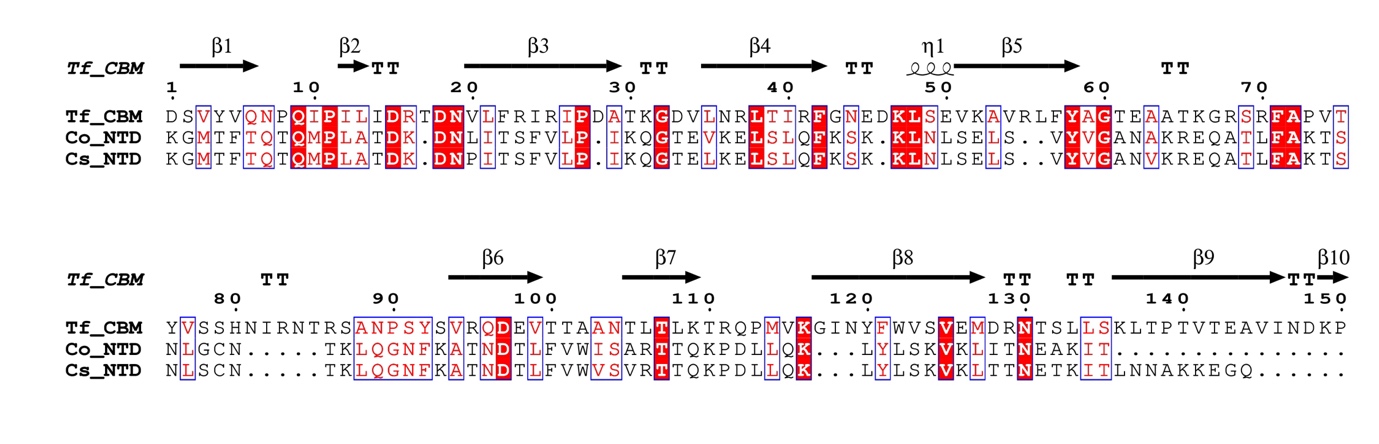
**

**Fig. S3. Multiple sequence alignment of the amino acid sequences of non-catalytic domain of sialidase homologues from *T. forsythia, C. ochracea* and *C. sputigena.*** Abbreviations: CBM: carbohydrate binding module, NTD: N-terminal domain. *T. forsythia* 92A.2 (Tf_CBM, WP_014225510.1); *C. ochracea* CCUG 9716 (Co_NTD, WP_012796827.1); *C. sputigena* CCUG 9714 (Cs_NTD, WP_002681834.1). The figure was prepared using ESPript 3.0^[1]^.

**
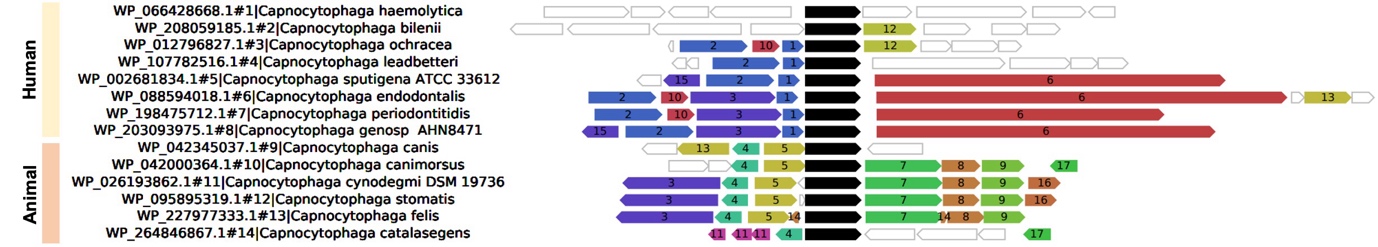
**

**Fig. S4. Gene conservation at the sialidase loci in *Capnocytophaga*.** Genes that encode proteins belonging to a homologous cluster (*i.e.*, proteins from the same family with the same predicted function) in more than one genomic neighborhood are colored and numbered the same. Non-conserved genes are uncolored. The sialidase gene is universally shown in black. Genes adjacent to the sialidase gene: Flanking gene 1 = alkylphosphonate utilization protein; Flanking gene 2 = molecular chaperone HtpG; Flanking gene 3 = outer membrane beta-barrel family protein; Flanking gene 4 = FadR/GntR family transcriptional regulator; Flanking gene 5 = AGE family epimerase/isomerase; Flanking gene 6 = T9SS type B sorting domain-containing protein; Flanking gene 7 = hypothetical protein; Flanking gene 8 = hypothetical protein; Flanking gene 9 = gliding motility-associated C-terminal domain-containing protein; Flanking gene 10 = EthD domain-containing protein; Flanking gene 11 = protein phosphatase 1 regulatory subunit 42; Flanking gene 12 = IMP dehydrogenase; Flanking gene 13 = tRNA (N6-isopentenyl adenosine(37)-C2)-methylthiotransferase MiaB; Flanking gene 14 = hypothetical protein; Flanking gene 15 = aspartate--ammonia ligase; Flanking gene 16 = DMT family transporter; Flanking gene 17 = DUF6261 family protein.

**
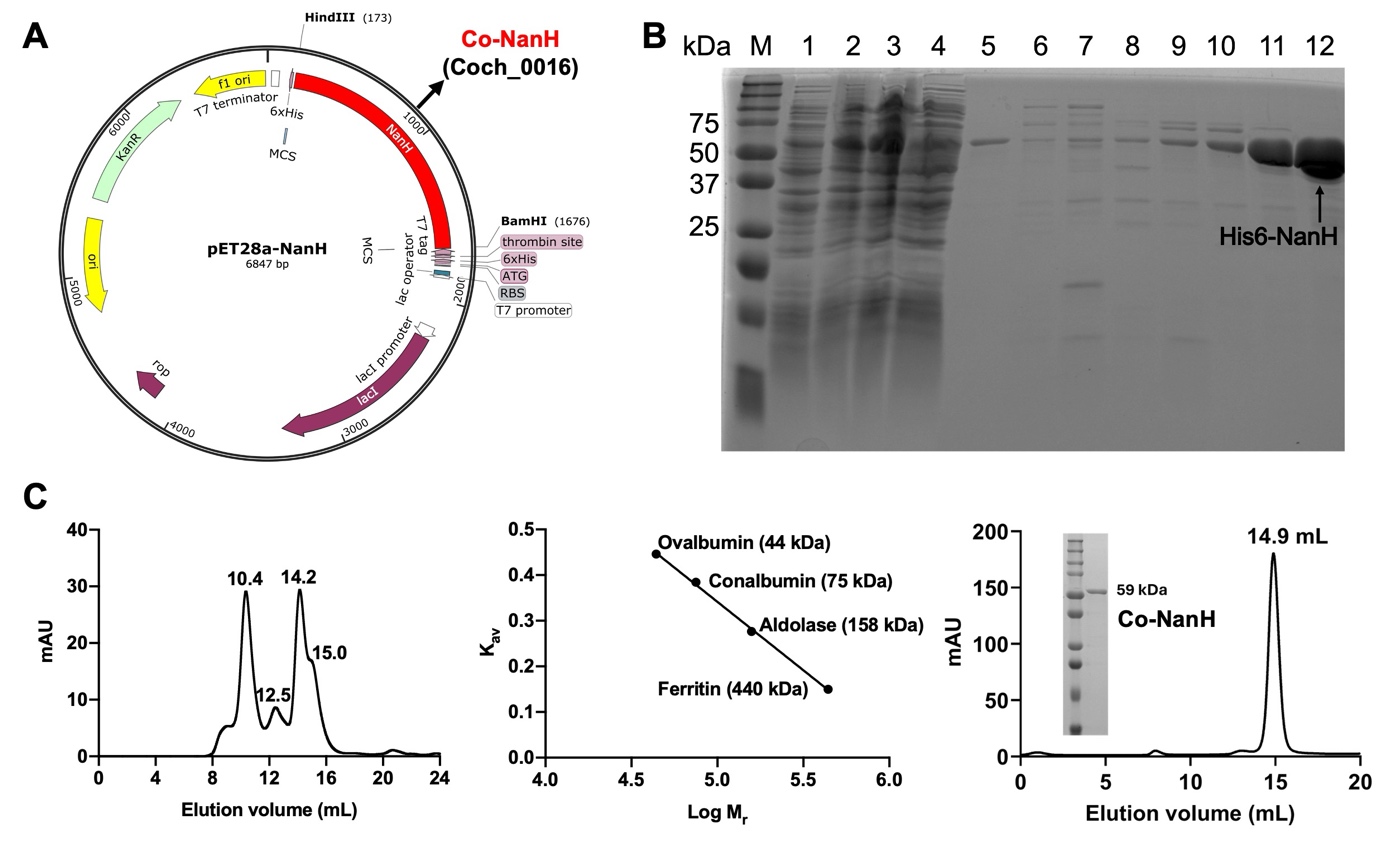
**

**Fig. S5. Purification of recombinant Co-NanH protein from *E. coli*.** (**A**) Construction of (**B**) SDS polyacrylamide gel electrophoresis of recombinant His6-NanH protein during purification. Proteins were separated by SDS-PAGE (12%) and visualized by Coomassie staining: M, standard molecular markers; lane 1, cell lysates before adding IPTG; lane 2, cell lysates after adding IPTG; lane 3, cell lysates; lane 4, Ni-NTA column eluate; lane 5, washed eluate after purification; lan6-lane12, Ni-NTA column eluate. (**C**) The size exclusion chromatograms of purified recombinant protein Co-NanH. The panel inset also shows the Coomassie brilliant blue (CBB) stained SDS-PAGE gel of the purified Co-NanH protein. Its elution volume was 14.9 mL, calculated apparent molecular weight *ca.* 58 kDa estimated multimericity: monomeric.

**
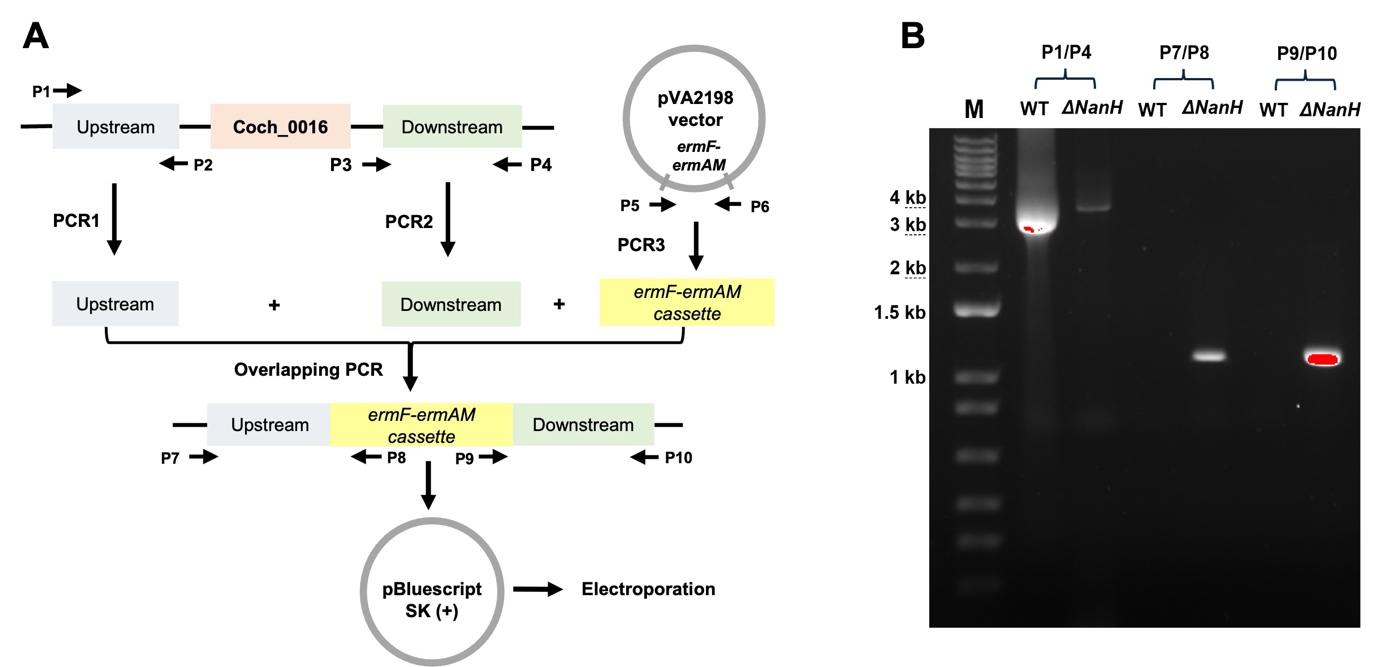
**

**Fig. S6. Schematic diagram of knockout plasmid construction and the knockout mutation verification.** (**A**) The diagram is a representative schematic of the generated chromosomal gene deletion construct used to create the sialidase gene deletion in the *C. ochracea* genetic background. The 5’ flanking region (upstream) was amplified from *C. ochracea* CCUG 9716 genomic DNA with primers P1 (including the restriction site) and P2 (PCR1). The 3’ flanking region (downstream) was amplified with P3 and P4 (including the restriction site) (PCR2). The primers P5 and P6 were used to amplify the *ermF-ermAM* resistance cassette (PCR3). The 5’ sequence of P2 and P3 corresponds to 20-nucleotide complementary to the 5’ terminus and 3’ terminus of the *ermF-ermAM* cassette, respectively. 100 ng of PCR1, PCR2 and PCR3 products were used in an ‘overlapping’ PCR reaction. The ‘overlapping’ PCR products were digested with the appropriate pair of restriction enzymes and ligated into pBluescript SK (+) plasmids for further electroporation. (**B**) Agarose gel image with the expected PCR product confirming the sialidase gene knockout.

**
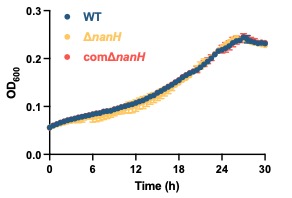
**

**Fig. S7. Growth curves of *C. ochracea* WT, Δ*nanH* and comΔ*nanH* strains.** Growth curve of *C. ochracea* WT, Δ*nanH* and comΔ*nanH* strains in the TSB.


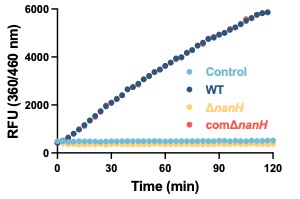


**Fig. S8. Sialidase activity in *C. ochracea* WT, Δ*nanH* and comΔ*nanH* strains.** The cell-associated sialidase activities in *C. ochracea* WT, Δ*nanH* and comΔ*nanH* strains were detected by MUNANA-based assay.

**Supplementary tables**

Table S1 Strains and plasmids

| Species and/or strains or plasmids | Description |
| --- | --- |
| Bacterial strains |  |
| *Escherichia coli* |  |
| DH10B | F-, *dcm*, *ompT*, *hsdS* (rB–, mB–), *gal*, λ(DE3) |
| BL21 (DE3) | F-, *mcrA*, Δ(*mrr*-*hsd*RMS-*mcr*BC), φ80*lacZ*ΔM15, ΔlacX74, *rec*A1, *end*A1, *ara*D139, Δ (ara, leu)7697, *gal*U, *gal*K, λ-, *rps*L, *nup*G |
| *Capnocytophaga ochracea* |  |
| CCUG 9716 | Human isolate, wild type |
| Δ*nanH* | Mutation of *nanH* by replacement of *ermF-ermAM* cassette |
| com-Δ*nanH* | Complementation of *nanH* in the genome |
| Plasmids |  |
| pET28a-Co-NanH | pET28a carrying full length Co-NanH gene from *C. ochracea* CCUG 9716 (cloned via BamHI/HindIII) |
| pVA2198 | 9.2-kb plasmid with the *ermF-ermAM* cassette, Ery^r^ |
| pBluescript SK (+) | 2.9-kb plasmid for cloning mutation PCR products; Amp^r^ |
| pT-COW | 1.0-kb plasmid for cloning complementation PCR products, Tc^r^ |

Antibiotic resistance phenotypes: Ery^r^, erythromycin resistant; Amp^r^, ampicillin resistant; Tc^r^: tetracycline resistance.

Table S2 Primers used in this study

| **Primers** | **Sequence (5’–3’)** |
| --- | --- |
| Co-NanH F | ATATATGGATCCATGCAAAAAGGAATGACTTT |
| Co-NanH R | ATATATAAGCTTTTACTTGATAAACTCTTTGAT |
| P1 | tcccccgggctgcaggaattcCTCTCTATATTTGCACATTGATTAATTGA |
| P2 | ctatcgggggtaccTTGTTAATTTATTGATTTAGAGATTGATTATTAA |
| P3 | taacaaGGTACCCCCGATAGCTTCCG |
| P4 | attcttttgctGGATCCCCGAAGCTGTCAG |
| P5 | cggggatccAGCAAAAGAATTTGTAACCTTAATAGAGA |
| P6 | ggtaccgggccccccctcgagCTACGGGGAGTTTTTCTACCTTACTG |
| P7 | GTAAAATCAATTATTTAAAAGTAGAGTTTTTATAA |
| P8 | AATACTTCTTGAGTTCAACTTATAAATGCAA |
| P9 | ATACCACAGATGTTCCAGATAAATATTGGAAGCT |
| P10 | CGCAAGCGCCCTAAGTTATCTTTGTTAGAAA |
| P11 | ATATATGGATCCATGCAAAAAGGAATGACTTTACCA |
| P12 | ATATATGTCGACTTACTTGATAAACTCTTTGAT |
| P13 | GGATTTGTAGAATGGAAGCCGGCGGCAC |
| P14 | TCTATCCAATTACACGGTGCCTGACTGC |

The sequences in lower case correspond to the sequence of the homology ‘arms’.

**Supplementary materials and methods**

**Webflag**

The gene neighborhood analysis of the sialidase homologues in the *Capnocytophaga* species was carried out with the online tool WebFlaGs^[2]^.

**Size exclusion chromatography**

Size exclusion chromatography was used to assess the multimeric state of purified recombinant proteins. It was performed on a Superdex 200 10/300 GL column (GE Healthcare) connected to an ÄKTA purifier system (GE Healthcare), using gel filtration buffer (25 mM Tris-HCl, pH 8.0, 500 mM NaCl, 1 mM EDTA, 5% glycerol), at a flow rate of 0.75 mL/min, monitoring the eluent at 280 nm. The protein molecular weight calibrants Ovalbumin (44 kDa), Conalbumin (75 kDa), Aldolase (158 kDa) and Ferritin (440 kDa) (GE Healthcare) were used to construct a calibration curve. The *Kav* values for the calibration kit proteins were calculated using the following equation: 𝐾𝑎𝑣 = $\frac{Ve-Vo}{Vc-Vo}$, *Ve* = elution volume, *Vo* = column void volume, and *Vc* =geometric column volume (Superdex 200 10/300 GL column: 24 mL). *Vo* was calculated by blue dextran solution (GE Healthcare). 0.5 mL protein sample was applied to the column using a 0.5 mL loading loop, determining *Ve*. The molecular weight of the purified recombinant protein was calculated using the calibration curve. In addition, eluted fractions were routinely analyzed by SDS-PAGE on 12% acrylamide/bisacrylamide gels.

**Construction of *C. ochracea* *nanH* mutant and complemented strains**

The insertional mutant of the *nanH* (Coch_0016) gene from *C. ochracea* CCUG 9716 was constructed by an allelic replacement strategy described previously^[3]^. Briefly, the respective 0.7 kb up/downstream fragments of the *nanH* gene and the *ermF-ermAM* cassette of the pVA2198 plasmid were PCR-amplified according to the schematic depicted in Fig. S6 using corresponding primers listed in Supplementary Table 2. The *ermF-ermAM* cassette was inserted between the upstream and downstream fragments of the sialidase gene by using an overlap extension PCR method. The resulting product was subjected to a final PCR amplification to generate a *ca.* 3.5-kb construct. The construct was cloned into pBluescript SK (+) (Stratagene), and the plasmids were transformed into *E. coli* DH5α. Transformants were selected on LB agar plates containing erythromycin (10 μg/mL) and ampicillin (100 μg/mL). Recombinant plasmid DNA was isolated using the QIAprep Spin Miniprep kit. After confirming its sequence via DNA sequencing, the recombinant plasmid DNA was digested with BsaI (NEB) and introduced into *C. ochracea* competent cells using electroporation. Transformant colonies that appear on the Columbia blood agar plates containing 10 μg/mL erythromycin were inoculated into 5 mL of reduced TSB containing 10 μg/mL erythromycin and incubated at 37°C under anaerobic conditions. After 3 days, 2 mL cultures were used to extract genomic DNA for mutant verification. The remaining 3 mL cultures were mixed with 5% sterilized glycerol and stored at -70°C. Δ*nanH* mutants were confirmed by detecting the correct junction of the *ermF-ermAM* cassette with the upstream and downstream of the sialidase gene, respectively, via PCR amplification by several pairs of primers and Sanger sequencing. After identifying the transformant corresponding to *nanH* deletion, 50 μL of the corresponding stock was subcultured into 5 mL reduced TSB with 10 μg/mL erythromycin and frozen for future use.

For complementation of *nanH* mutations (com-Δ*nanH*), the procedure is generally identical to the construction of the Δ*nanH* mutant. In brief, the *nanH* gene was PCR-amplified from *C. ochracea* CCUG 9716 chromosomal DNA using corresponding primers (P11 and P12) listed in Supplementary Table 2 and the DNA fragment was inserted into pT-COW plasmid^[4,5]^. Clones were confirmed via DNA sequencing. Purified recombinant plasmid DNA was transformed into Δ*nanH* mutant competent cells via electroporation. Transformants with the pT-COW-*nanH* plasmid were selected by TSB agar plates with tetracycline.

**Supplementary References**

1. Robert, X., & Gouet, P. (2014). Deciphering key features in protein structures with the new ENDscript server. *Nucleic Acids Research*, *42*(W1), W320–W324.

2. Saha, C. K., Sanches Pires, R., Brolin, H., Delannoy, M., & Atkinson, G. C. (2021). FlaGs and webFlaGs: Discovering novel biology through the analysis of gene neighbourhood conservation. *Bioinformatics (Oxford, England)*, *37*(9), 1312–1314.

3. Mally, M., & Cornelis, G. R. (2008). Genetic tools for studying Capnocytophaga canimorsus. *Applied and Environmental Microbiology*, *74*(20), 6369–6377.

4. Rothenberger, C. M., Yu, M., Kim, H.-M., Cheung, Y.-W., Chang, Y.-W., & Davey, M. E. (2024). An outer membrane vesicle specific lipoprotein promotes Porphyromonas gingivalis aggregation on red blood cells. *Current Research in Microbial Sciences*, *7*, 100249.

5. Gardner, R. G., Russell, J. B., Wilson, D. B., Wang, G. R., & Shoemaker, N. B. (1996). Use of a modified Bacteroides-Prevotella shuttle vector to transfer a reconstructed beta-1,4-D-endoglucanase gene into Bacteroides uniformis and Prevotella ruminicola B(1)4. *Applied and Environmental Microbiology*, *62*(1), 196–202.
